# Seven genome sequences of airborne, bacterial isolates from Antarctica

**DOI:** 10.1128/mra.01129-23

**Published:** 2024-05-15

**Authors:** Markus Dieser, Heidi J. Smith, Christine M. Foreman

**Affiliations:** 1 Center for Biofilm Engineering, Montana State University, Bozeman, Montana, USA; 2 Department of Chemical and Biological Engineering, Montana State University, Bozeman, Montana, USA; 3 Department of Microbiology and Cell Biology, Montana State University, Bozeman, Montana, USA; University of Southern California, Los Angeles, California, USA

**Keywords:** Antarctica, aeolian transport, glaciers

## Abstract

Ice-covered and remote landscapes in the McMurdo Dry Valleys, Antarctica, are likely seeded by aeolian transport of biological material from ice-free local or distant environments. Here, we report the genome sequences of seven bacteria isolated from aerosols collected on top of two dry valley glaciers.

## ANNOUNCEMENT

Aeolian transport disperses microorganisms between and across lands and waters (1). While the primary source of seeding Antarctica is still open to question (2), both local and long-distance routes have been highlighted (3–6). Once deposited, different environments will exert strong selective pressure (7).

Of the seven bacterial genome sequences reported herein, six were classified as *Arthrobacter* species. *Arthrobacter* spp. are commonly found in soils (8), including Antarctica (9, 10). *Arthrobacter* spp. can grow on a large variety of substrates and are extremely resistant to drying and starvation (8). Resistance to oxygen radicals and osmotic stress may enhance their ability to overcome environmental stresses (9–11). Genome comparisons to other *Arthrobacter* spp. can identify relevant traits to both the local and global distribution of this genus (9–14). Moreover, such comparisons can help address the likely source of these isolates and determine whether airborne microbes have genetic traits that can elevate their chance of successfully colonizing a new habitat.

Air samples were collected from two locations within Victoria Land, Antarctica (Canada Glacier 77°37′S 162°59′E; Cotton Glacier 77°07′S 161°40′E) with a Sartorius MD8 portable air sampler onto 80 mm, 3 µm Sartorius Gelatin Filter units. The filters were transferred onto R2A agar plates. Bacteria were incubated and isolated on R2A plates at 4°C. The cetyltrimethylammonium bromide procedure was used for extracting DNA from bacterial isolates grown to late exponential phase in R2A broth at 4°C while shaking at 150 rpm (15). Standard quality shotgun libraries were sequenced on the Illumina NovaSeq S4 platform (16). Libraries were prepared on a PerkinElmer sciclone NGS robotic liquid handling workstation using the Kapa Biosystems library kit. DNA was sheared to 459 bp using a Covaris LE220 focused-ultrasonicator. DNA fragments were size selected by double-SPRI. Fragments were end-repaired, A-tailed, and ligated with Illumina-compatible sequencing adaptors. Raw Illumina sequences were quality filtered using BBTools (17) per SOP 1061 and assembled with SPAdes (≥version v3.14.1; –phred-offset 33 –cov-cutoff auto -t 16 -m 64 –careful -k 25,55,95) (18). Contigs with a length < 1 kb (BBTools reformat.sh: minlength = 1000 ow = t) were discarded. CRISPR elements were identified using the programs CRT (CRISPR Recognition Tool) (19) and PILER-CR (20). All genomes were annotated in the Integrated Microbial Genomes (IMG) database (≥IMG Annotation Pipeline v.5.0.19) (21). Genome completeness and contamination were estimated using checkM (22). The genome-sequence-acquired 16S ribosomal RNA gene was queried in the command line against the SILVA database 138.1 with blastn, from BLAST + 2.13.0 for taxonomic classification (23). Sequence details are given in Table 1.

**TABLE 1:** GenBank accession numbers and summary of genome characteristics^*a*^

Organism (% match to genus) accession number	Coverage (×)	# contigs	L50/N50 (bp)	Size (bp)	% C/C	GC (%)^*c*^	# genes	# C
*Arthrobacter* sp. CAN_A1 (99.9) JADOUR000000000 SRR13164829	353.5	43	5/279,561	3,377,403	99.08/0.00	64.34	3,315	–^*b*^
*Arthrobacter* sp. CAN_A2 (99.5) JADOUS000000000 SRR13291739	453.6	79	5/314,970	3,587,188	99.08/0.00	69.17	3,428	1
*Arthrobacter* sp. CAN_A212 (99.9) JADOUT000000000 SRR13291872	314.3	79	8/132,108	3,560,477	99.54/0.11	64.23	3,491	–
*Arthrobacter* sp. CAN_A214 (98.6) JADOUU000000000 SRR13291917	359.2	99	7/150,747	3,753,103	99.54/2.16	64.86	3,670	–
*Arthrobacter* sp. CAN_A6 (98.6) JADOUV000000000 SRR13292022	299.6	48	4/291,372	3,710,640	99.54/0.63	65.19	3,625	–
*Pedobacter* sp. CAN_A7 (97.8) JADOUW000000000 SRR13292021	183.3	10	2/1,232,302	4,147,385	97.93/0.48	40.80	3,674	1
*Arthrobacter* sp. CG_A4 (99.6) JAXCID000000000 SRR25764252	365.5	107	17/85,381	4,169,606	99.42/0.00	66.40	3,971	–

^*a*^ C/C, completeness/contamination estimate; C, CRISPR.

^*b*^ –, none.

^*c*^ GC, guanine-cytosine content.

## Data Availability

Sequence read archive data are available through the NCBI server. Accession numbers are listed in Table 1.
